# Supplementary material for: Improving Multi-Delay-ASL through specialized reconstruction
Source: ArXiv. 2026 Sep 25:arXiv:2609.30923v1. Preprint. [Version 1] (PMC13622988)
Supplement: Supplementary file 1 [file nihpp2609.30923v1-supplement-1.pdf]

B Additional Data

B.1 Subject 1 - PWIs - Denoised

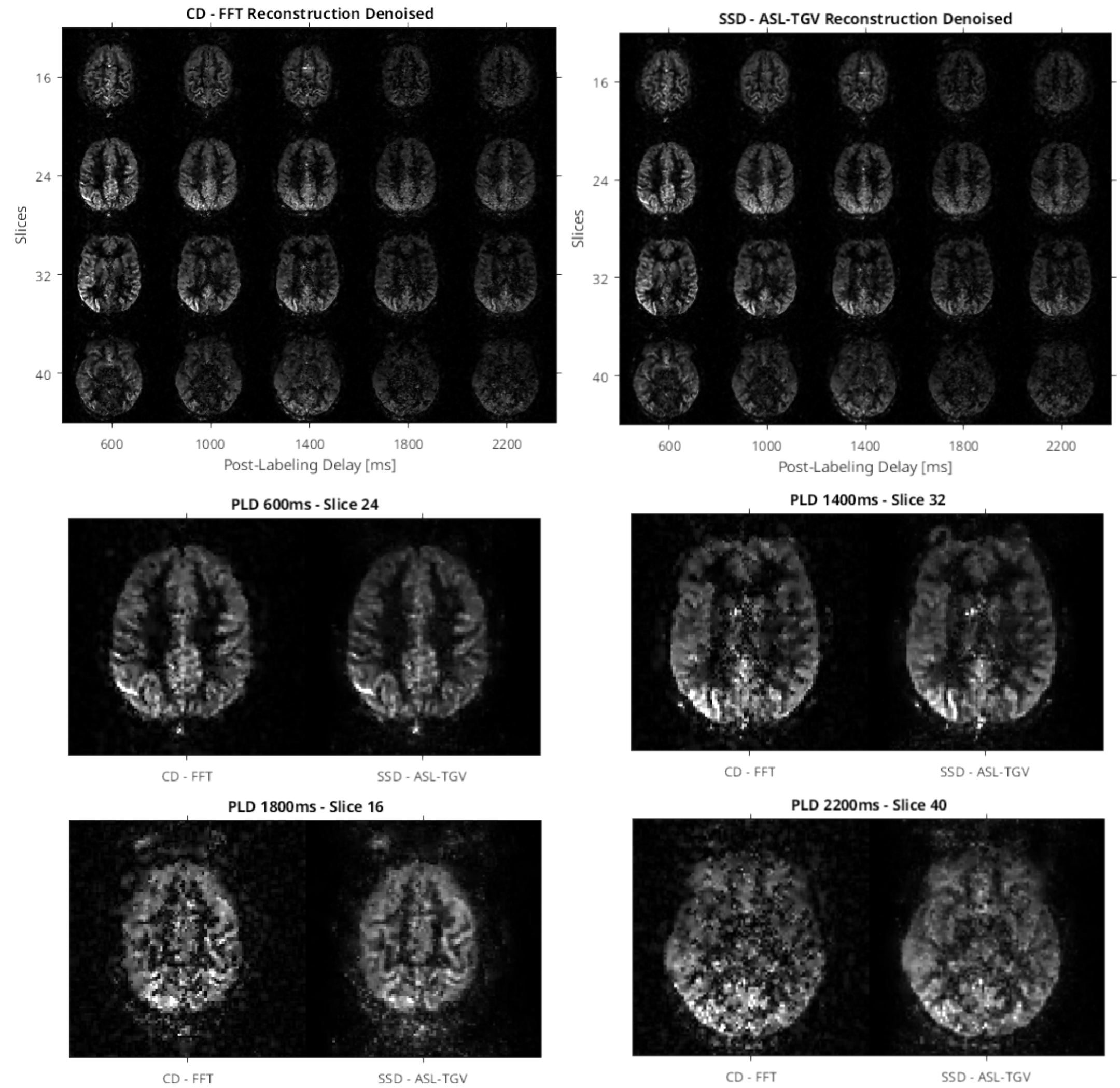

Figure S1: These are the denoised images created with the two different reconstructions for the first visit of subject 1. The top row shows four selected slices for each PLD for both reconstructions. Below are direct comparisons of the two reconstructions for the selected slices at one of the PLDs.

B.2 Subject 21 - PWIs and CBF - Outlier

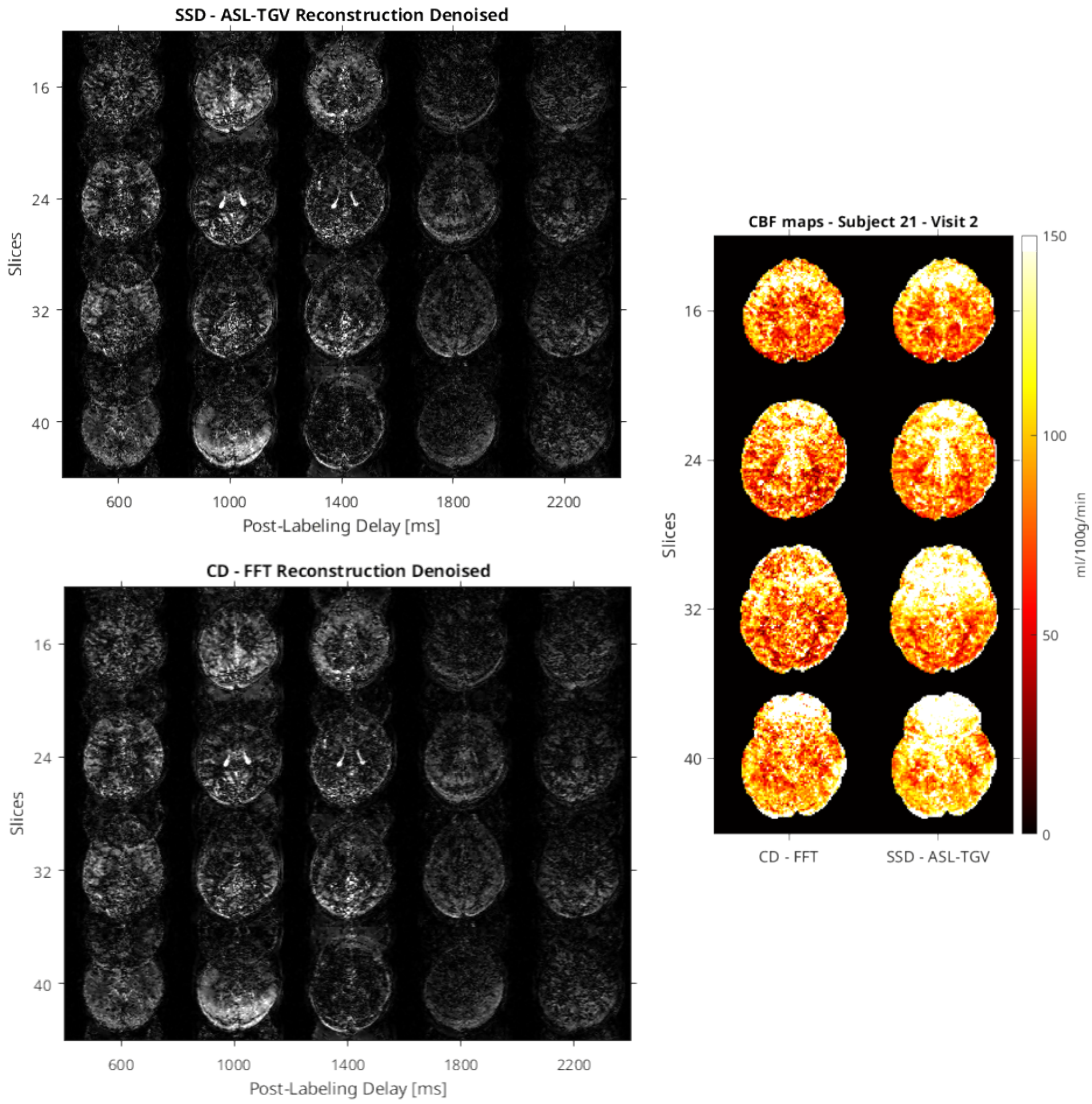

Figure S2: The images and CBF map of visit 2 of subject 21. The artifacts in the image produce a highly irregular CBF map. This subject was thus excluded from the test-retest evaluation.
